# Dermal and neural toxicity caused by acrylamide exposure in two Korean grouting workers: a case report

**DOI:** 10.1186/s40557-017-0207-7

**Published:** 2017-10-09

**Authors:** Heeyun Kim, Sang Gil Lee, Jeongbae Rhie

**Affiliations:** 1 Department of Occupational and Environmental Medicine, Kangbuk Samsung Hospital, Sungkyunkwan University School of Medicine, Seoul, Republic of Korea; 2 Occupational Safety and Health Research Institute, KOSHA, Republic of Korea, Ulsan, Republic of Korea; 3 Department of Occupational and Environmental Medicine, Dankook University College of Medicine, Cheonan, 201 Manghyang-ro, Dongnam-gu, Cheonan-si, Chungcheongnam-do 31116 Republic of Korea

**Keywords:** Acrylamide, Neuropathy, Grouting agent, Dermatitis, Cerebellar ataxia, Korea

## Abstract

**Background:**

Peripheral neuritis caused by acrylamide is well-known, and many Korean grouting workers are frequently exposed to acrylamide in grouting agents that are injected into cracked concrete. We recently encountered two cases of dermal and neural toxicity in Korean grouting workers with exposure to grouting agents that contained a high concentration of acrylamide.

**Case presentation:**

The first case involved a 44-year-old man with 8 years of waterproofing experience. The patient developed peeling skin on both hands while grouting, which progressed to systemic neurological symptoms, such as reduced sensory function and strength. The patient was diagnosed with peripheral neuropathy caused by acrylamide exposure, and fully recovered after conservative treatment and withdrawal of exposure to the grouting agent. The second case involved a 34-year-old man with 10 years of grouting experience. The patient initially experienced weakness in both legs, which progressed to weakness in his arms and uncontrolled phonation. After being hospitalized, he was diagnosed with cerebellar ataxia and peripheral neuropathy caused by acrylamide exposure, and was discharged after conservative treatment. Our follow-up investigation revealed that both workers were recently exposed to grouting agents that contained higher concentrations of acrylamide, compared to the agents that they had previously been using.

**Conclusions:**

Both workers had workplace acrylamide exposure through dermal contact plus inhalation of dust and vapor, which led to the neural toxicity and dermatitis. Therefore, government studies are needed to investigate the current status of workplace acrylamide use, and to protect workers from the hazardous effects of using acrylamide-containing grouting agents.

## Background

Workers apply grouting agents to cracks in concrete in order to prevent water seeping into floors, roofs, and/or walls. There are various types of grouting agents, and acrylamide-based grouting agents have several advantages, which include their relatively low viscosity, high stability, and more predictable gelation time. The acrylamide monomer (C_3_H_5_NO, molecular weight: 71.08) provides waterproofing protection when it has polymerized. Acrylamide is a colorless and odorless solid that is highly soluble in water, and is frequently used as a flocculant, textile treatment agent, dispersant, paper strengthener, and adhesive [1].

There are many reported cases of neurological disorders among workers who were exposed to an acrylamide-containing grouting agent. One report described a case of multiple neuropathy in a 30-year-old man who was performing acrylamide production in Korea [2]. Peripheral neuropathy after exposure to an acrylamide-containing grouting agent has also been reported at a UK construction site in 1977 (6 workers), in the Chinese city of Sinsang in 1994 (41 workers), and in a Norwegian tunnel site in 2004 (24 workers) [3–5]. The American Environmental Protection Agency issued a warning in 1987 regarding the hazards of airborne exposure and dermal contact with acrylamide during chemical grouting work [6], and the European Union has also recommended limiting the use of these agents.

The 0.1% limit for acrylamide includes other sources of free acrylamide that are used in the grouting process, such as N-methylolacrylamide [7]. Nevertheless, acrylamide is actively used in many industrial sites throughout the world, and the benefits of acrylamide have led to its persistence in grouting work. Furthermore, acrylamide is frequently used in Korea for grouting work, and there does not appear to be significant interest in the prevalence of its use or its effects on worker health. Therefore, we report two cases of dermal and neural toxicity that involved grouting workers with workplace acrylamide exposure. We hope that these cases will draw attention to the health risks of workers who use grouting agents with high concentrations of acrylamide.

## Case presentation

### Patient 1

The first case involved a 44-year-old married Korean man who experienced skin peeling, edema, redness on both hands, and erectile dysfunction after switching grouting agents at work (near the end of June 2014). Between December 2014 and early January 2015, the patient experienced deteriorating sensory function and numbness in his hands and feet, as well as decreased arm and leg strength. The patient eventually found it difficult to climb stairs, and collapsed on January 2, 2015. Thus, he was admitted to a local neurology department at a university hospital.

At the admission, the patient reported having no specific medical history, family history, or history of viral infection (e.g., influenza). The patient also reported not having any specific hobbies, drug habits, or alcohol consumption, although he reported a 25 pack-year smoking habit. He had performed grouting work for 8 years and had been working at the same repair firm since 2009, where he injected grouting agents into cracks in concrete ceilings (7 h/day, 5 days/week). The patient reported wearing cotton gloves and a dust mask, although he admitted that he often worked without the mask. His previous jobs included electrical wiring (7 years), running a bike shop (3 years), commercial taxi driving, delivery truck driving, and sales and operations at a pipe manufacturer. We judged it unlikely that his symptoms had been caused by workplace exposure at those jobs.

### Physical examination

The patient underwent a neurological examination at the hospital on January 2, 2015, which revealed poor whole-body muscular strength (less than grade 2: unable to move shoulders, elbows, wrists, and hands when gravity is eliminated). Lower-body testing also revealed weakness in his hips, knees, ankles, and large toes. At the examination, the patient could not walk, sit, or stand without assistance. A sensory evaluation revealed normalcy in his upper and lower body, although the patient complained of numbness at the ends of his fingers and toes. The deep tendon reflex test did not elicit a biceps reflex or Babinski’s reflex on either side. Other cerebellar and central nervous system tests revealed no specific irregularities, and the patient was fully conscious.

### Diagnostic assessment

Immediately after the physical examination, the patient underwent lower-body electromyography, which revealed normal motor nerve conduction velocity (NCV) in both posterior tibial nerves and reduced compound muscle action potential (CMAP) amplitudes at the left and right tibia. Thus, bilateral tibial neuropathy was suspected with extended H-reflex on both sides, and the reduced CMAP indicated severe nerve conduction dysfunction. Nerve conduction testing was repeated on the fourth day of the hospitalization, which revealed extension of the terminal latency, F-wave latency in the right median nerve, reduced CMAP amplitude and motor NCV, and extension of the right ulnar nerve’s F-wave latency. These findings suggested that the patient had sensorimotor polyneuropathy.

### Intervention and outcome

Acute inflammatory demyelinating polyneuropathy was suspected on the fifth day of the patient’s hospitalization, and the patient was treated using intravenous immunoglobulin and IgA. After hospitalization (i.e., withdrawal of acrylamide exposure) and conservative treatment, the patient’s symptoms improved and his muscle weakness and sensory abnormalities generally resolved. On March 16, 2015, nerve conduction testing was performed at the neurology department’s outpatient clinic, which revealed normal motor NCV in both posterior tibial nerves, a reduced CMAP amplitude at the right knee, and otherwise normal findings. These results were markedly better than the results from the patient’s admission, and a follow-up in June 2016 revealed that the patient had regained normal sensory functions.

### Patient 2

The second case involved a 34-year-old Korean man who reported experiencing sudden weakness in both legs at his worksite on July 11, 2014. Two days after the first episode of bilateral leg weakness, the patient also experienced weakness in both hands and subsequently slurred speech. The patient was admitted to a university hospital during July 18–29 because of ataxia and weakness. The patient was very obese, single, and did not report any significant medical or family history. There were no signs of infectious diseases in the laboratory and physical examinations. The patient did not report having any specific hobbies or drug use, although he reported a 20 pack-year smoking habit and a social drinking habit. The patient reported performing grouting work at the same company for the past 10 years, and also noted that his leg weakness and slurred speech had coincided with switching to a new grouting agent (DK Acryil AA; June 2 to July 12, 2014).

### Physical examination

The patient underwent a physical examination on July 18, which revealed an ataxic gait and normal bodily strength. Functional testing (finger-to-nose and heel-to-shin) confirmed bilateral ataxia and dysdiadochokinesia. Romberg’s sign (swaying with his eyes open) was also observed, and the patient had brownish scaly patches on his hands and knees. Sensory nerve tests revealed position and vibration impairments, with dorsal column involvement, although normal findings were observed for light touch, pain, and temperature. Thus, the patient was diagnosed with cerebellar ataxia and peripheral neuropathy.

### Diagnostic assessment

On July 18, brain magnetic resonance imaging revealed no specific findings. On July 23, brain single-photon emission computed tomography revealed possible focal hypoperfusion in the left parietal cortex and diffuse hypoperfusion in the bilateral temporal cortices and right thalamus. Nerve conduction testing revealed delayed terminal latencies, normal amplitudes on the median and ulnar nerves, and normal CMAPs on the peroneal and posterior tibial nerves, which suggested that the patient had demyelinating predominantly sensorimotor polyneuropathy. Sensory nerve conduction testing revealed slow conduction velocities in both median nerves, normal sensory nerve action potentials (SNAPs) on both ulnar nerves, normal SNAPs on the sural and superficial peroneal nerves, and delayed F-wave latencies in the median and ulnar nerves.

### Intervention and outcome

The patient received a 12-day conservative treatment using analgesics (such as tramadol), subsequently recovered, and was discharged on July 29, 2014. An outpatient visit on August 13 revealed persistent ataxia, although his phonation was controlled. Another follow-up on September 18 revealed no ataxia or motor impairment. On November 8, right wrist median sensorimotor neuropathy was observed during nerve conduction testing, although improvements were observed in the median and right ulnar nerve terminal latencies, CMAPs, NCVs, and SNAPs, as well as bilateral improvements in the F-wave and H reflex (vs. the tests from July 2014).

### Exposure assessment

The patient had repaired ceiling cracks in underground parking garages by injection grouting solutions into the cracked concrete. The patient worked for 7 h per day (9 AM to 5 PM with a 1-h lunch break), 5 days per week, as part of a 3-person team. The patient’s primary duty involved grinding the leaking section and applying the grouting solution using an injector, and the other members checked the mixing status or adjusted the injector, which sat beside the patient. Before the incident, the patient had used a transparent grouting agent (EA-3000 Acrylic Waterproof Material), although the agent was switched to DK Acryil AA (D Infrastructure) in June 2014. The DK Acryil AA compound is mixed and applied, and produces heat, steam, and a strong irritating odor as it cures. The underground parking garages were typically unventilated, and the gases from the work would have contacted the patient’s respiratory and dermal systems.

**Fig. 1:**
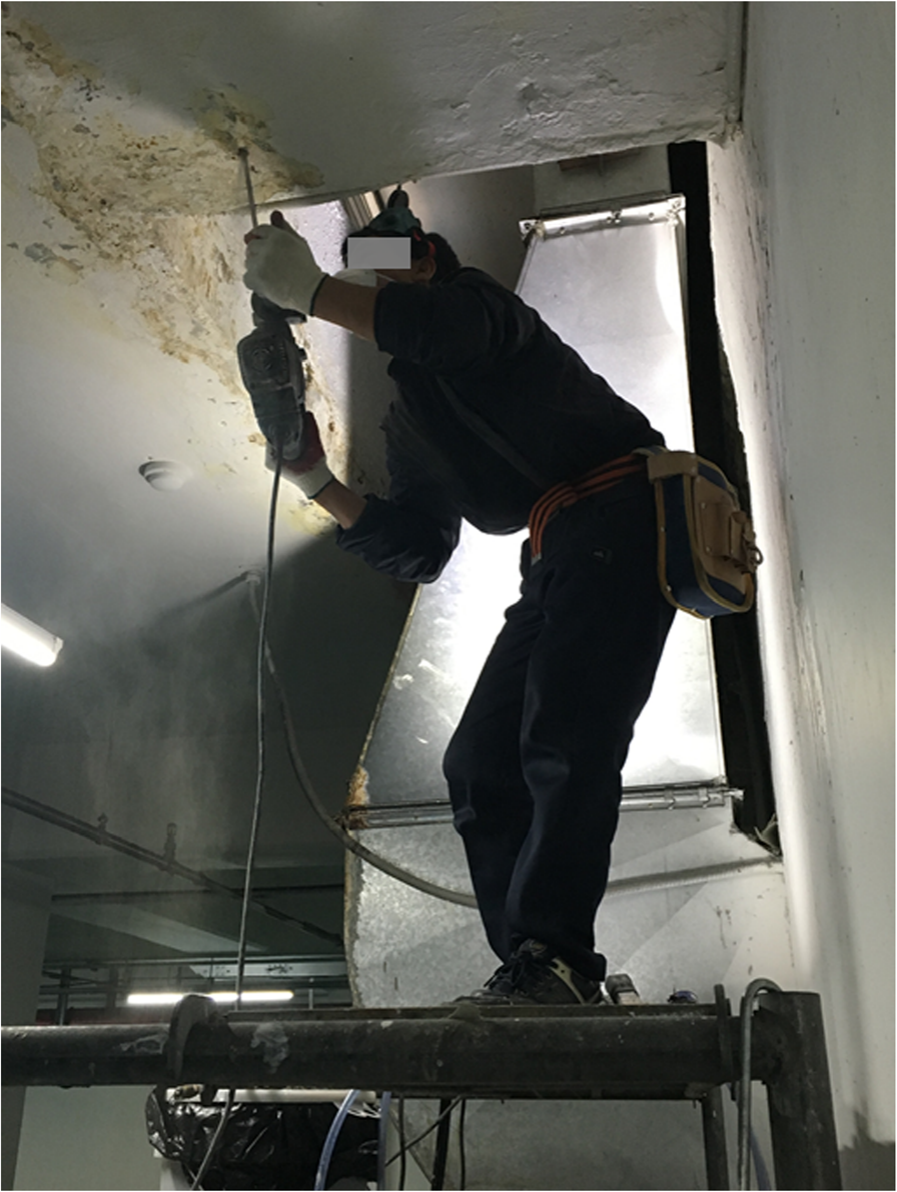
Patient 1 performs ceiling work and injects grouting

**Fig. 2:**
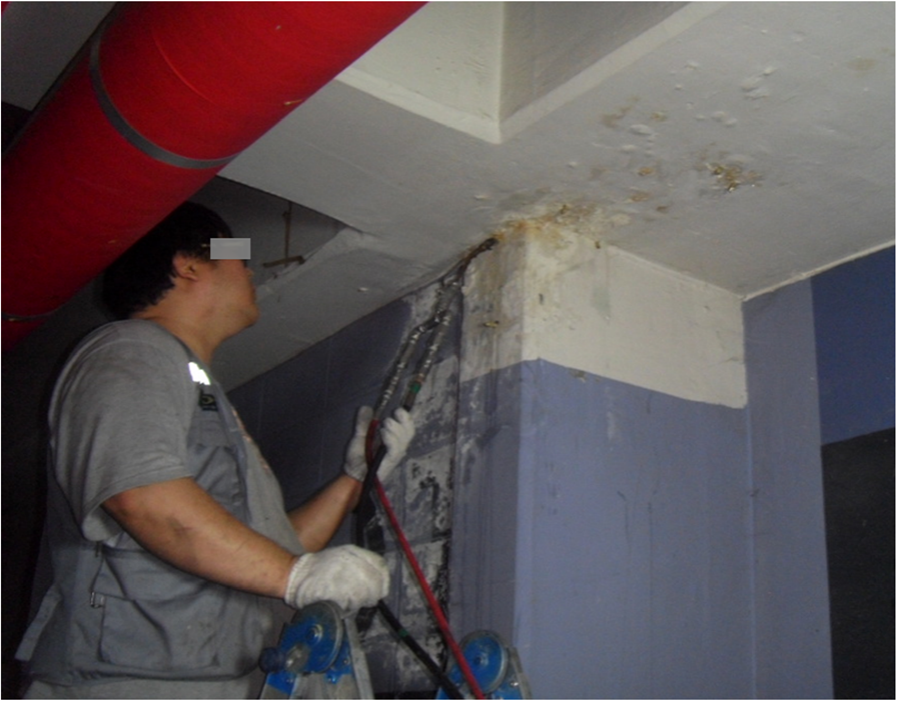
Patient 2 performs ceiling work and injects grouting

To inject the grouting solutions into the cracked concrete wall, the patient had to hold the applicator’s nozzle and was frequently exposed to splash-back (5–7 times/day), which resulted in the agent contacting his hands, face, and other exposed body parts. Thus, the patient frequently worked with the agent smeared on his skin (Figs. 1 and 2). The patient was unwilling to stop working after these exposures, as the agent would have hardened in the applicator nozzle and tubing. The patient’s protective equipment consisted of semi-coated cotton gloves and a dust mask when the garage’s ventilation system was inoperable. The workers occasionally wore the dust masks when the odor became unmanageable, although they reported frequently not wearing the masks because they were uncomfortable. The workers were not aware of the hazardous effects of acrylamide-containing agents on their skin, and the patients did not wash the agents from his exposed skin with water after completing his work.

We performed raw materials analysis using a commercially available grouting agent and additives from Epoxy Korea, as well as a sample of the grouting agent and additives that the worker had stored since June 2014 (when his symptoms developed). The grouting agent that the patient had been using was 39% *w*/w acrylamide(DK Acryil AA) and 32% w/w acrylamide(EA-3000) (Table 1).

**Table 1:** Acrylamide concentrates from grouting agents that were used by the patients

Grouting agent	Acrylamide monomer
DK Acryil AA (D Infrastructure)	39% *w*/w acrylamide
EA-3000 Acrylic Waterproof Material	32% w/w acrylamide

Analyzed using the Agilent 5973 CG-MSD device

## Discussion

The present report describes our experience with two grouting workers who were exposed to acrylamide and subsequently developed dermal and neural toxicity. Both patients had experienced dermal and respiratory exposure, although their clinical features were different. Patient 1 experienced skin peeling at 2 weeks and systemic neuropathy at 6 months after exposure to an agent with a high concentration of acrylamide. Patient 2 experienced cerebellar dysfunctions, including gate ataxia and slurred speech, at 1 month after exposure to a similar agent. Both workers experienced improvement after conservative treatment and elimination of the acrylamide exposure during their hospitalization.

Workers may be exposed to acrylamide through ingestion, skin contact, or vapor/dust inhalation [8]. The health risks of dermal exposure to acrylamide are well-known, and the Association Advancing Occupational and Environmental Health has published related warnings in the TLV/BEI guidelines since 1985. In addition, that association reported in 2004 that acrylamide can be absorbed when it is present in steam, and established acrylamide as a target for active control [9]. Furthermore, in 2009, the National Institute for Occupational Safety and Health also confirmed that dermal acrylamide exposure is a serious issue, as they found that the SI ratio (ratio of skin to inhalation doses) was 2955; an SI ratio of >0.1 indicates a risk of systemic toxicity after skin exposure [10].

Exposure to acrylamide can occur through the skin and possibly vapor inhalation, which can be caused by the polymerization process during work with monomeric acrylamide [11]. Monomeric acrylamide is neurotoxic and probably carcinogenic to humans. Although monomeric acrylamide is easily polymerized to an insoluble polymer compound using heat or catalysts, newly synthesized polymer compounds still contain 0.05–5.0% monomeric acrylamide [12]. After exposure, acrylamide is rapidly distributed through the bloodstream and 10% binds to red blood cells. The initial dose has a half-life of approximately 2 h and causes significant neurological effects, although it does not collect in the nervous system [13]. The effects of acrylamide can be categorized as local (on the skin) and systemic (in the nervous system). Dermal exposure may result in an exfoliative reddish rash in humans. Acrylamide neurotoxicity in the peripheral and central nervous system could damage the nerve terminals through membrane fusion mechanisms and tubulovesicular alterations [11]. Peripheral neuropathy is the most common neurological effect, and the peripheral nerve terminals are a primary site of acrylamide action, with possible inhibition of membrane-fusion processes impairing neurotransmitter release. The sensory abnormalities are typically related to sense of location, temperature, and vibration. The most common neurological symptoms are loss of balance and gait ataxia, although neurological testing may also reveal loss of deep tissue reflexes, muscle contraction, and Romberg’s signs [14–16]. Cerebral ataxia has been reported among workers at a UK factory and among Australian miners in 1967 [17]. In 1989, 71 Chinese factory workers developed chronic neuropathy after prolonged exposure to liquid acrylamide, and three of these patients developed cerebral ataxia [18].

Symptom progression after acrylamide exposure is an important consideration in the diagnosis of workers with multiple peripheral neuropathies. Low-level exposure to acrylamide could also induce ataxia, gait abnormalities, weakness, dermal abnormalities, and extremity numbness. Previous studies have demonstrated that acrylamide exposure could aggravate paralysis of the cerebrospinal system and irritate the eyes and skin [19, 20].

Acrylamide can induce neurotoxicity through multiple exposure routes, and although the precise toxic mechanism(s) remain unclear, the cumulative effect is generally neurotoxicity [11]. There is typically a prolonged latency period between the exposure and symptom development, which is likely related to the fact that nerve damage must exceed a threshold before neurological symptoms become apparent. In most cases, the symptoms and signs of acrylamide exposure have been reversible, with full resolution after 2–12 months of exposure withdrawal, although some symptoms can persist for several years [5]. A full recovery may take months to years after the exposure has been eliminated, as axons regenerate at a rate of 2–3 mm per day and the recovery begins at the nerve’s proximal end and progresses towards the distal end [21]. Therefore, considering the clinical symptoms and latency period can provide important information.

In cases with continuous acrylamide exposure, progressive retrograde degeneration of the distal axon regions can occur without degeneration of the proximal segments [22]. Axonal degeneration caused by acrylamide exposure is characterized by the reduction of motor or sensory amplitudes during nerve conduction tests. One possible mechanism is weakness of the nerve endings, especially between the nerve terminals and blood vessels, and another possible mechanism is a toxic effect on neuronal metabolism and protein synthesis. Previous studies have demonstrated that acrylamide can damage both motor and sensory nerve fibers, especially long or sensory fibers [23]. Patient 1 exhibited extension of the terminal latency, F-wave latency in the right median nerve, and normal motor NCV in both posterior tibial nerves, while Patient 2 exhibited delayed terminal latencies, normal amplitudes on the median and ulnar nerves, and normal CMAPs on the peroneal and posterior tibial nerves. The clinical interpretation of the nerve conduction test results suggested myelopathy, rather than axonopathy that was related to typical neural toxicity from acrylamide exposure. These results could indicate a toxic effect on myelin that reduced upper-limb NCV, with only a slightly significant reduction in the sensory amplitude of the median nerve. This finding conflicts with the results of previous studies regarding acrylamide exposure [18], which have consistently described signs of axonal degeneration with reduced amplitudes [5]. One explanation may be that direct contact after diffusion through the skin of the hands may have caused myelinolytic effects on the distal nerve fibers, which differed from the toxic effects on neuron metabolism that caused axonal damage in the previous studies. However, the present patients exhibited an apparent reduction in the mean sensory amplitude and NCVs of the sural nerve between 1 and 6 months after the acrylamide exposure. The type and localization of this lesion is similar to other examples of acrylamide’s effects on humans, and this delayed finding suggests that the changes may have taken a relatively long time to develop and also persisted for an unexpectedly long time [5].

The Korean Ministry of Labor has proposed that acrylamide exposure be limited to <0.03 mg/m^3^ and has recommended taking precautions to prevent skin absorption. This is the same exposure standard that is recommended by the National Institute for Occupational Safety and Health and the American Conference of Governmental Industrial Hygienists. However, Korean workers, including both patients in this report, may have higher exposures, compared to workers in other countries with the same exposure limits. The American Environmental Protection Agency has estimated that respiratory and dermal exposure levels were 0.008–0.12 mg/m^3^ and 0.6–5.0 mg/h, respectively, based on a TLV-TWA value of 0.008–0.12 mg/m^3^ and an acrylamide concentration of 5% [24]. However the two patients had been working with even higher concentrations of acrylamide (39%). Thus, their dermal and neural toxic effects may have been related to high levels of respiratory and dermal exposure that were caused by the high concentrations of the acrylamide in the grouting agents. Although there is some regulation of the production of acrylamide-containing grouting agents in Korea, the actual application of these agents is performed by a fragmented group of small-size of enterprises, which has led to insufficient oversight and management among grouting workers.

The Korean Occupational Safety and Health Agency has recognized the need for a management or control system to protect workers who are exposed to high concentrations of acrylamide in the workplace. In the European Union, acrylamide has been replaced by less toxic alternatives to acrylamide, such as N-methylolacrylamide. Thus, alternatives to acrylamide are needed in Korea, especially for employees of small waterproofing businesses [25]. Government oversight and regulation of acrylamide use remain important, and there are several related recommendations, such as adequate ventilation to control dust and gas, wearing appropriate personal protective equipment (e.g., a respirator, facial shielding, and impermeable protective gloves), and washing contaminated skin after work. Therefore, it is important to educate Korean grouting workers regarding exposure to acrylamide and the associated health risks.

## Conclusion

In conclusion, there is a general lack of interest in the management of grouting workers’ health, which is further exacerbated by the requirement to work quickly to prevent clogging of the applicator. Therefore, stronger control of grouting agents is needed, as well as education and measures to protect workers’ health. These issues are further magnified because of the high exposure levels and the frequency of splash-back in this industry.

## Abbreviations

CMAP: Compound muscle action potential
NCV: Nerve conduction velocity
SNAP: Sensory nerve action potentials
